# Assessing discriminative ability of risk models in clustered data

**DOI:** 10.1186/1471-2288-14-5

**Published:** 2014-01-15

**Authors:** David van Klaveren, Ewout W Steyerberg, Pablo Perel, Yvonne Vergouwe

**Affiliations:** 1 Department of Public Health, Erasmus MC, Dr. Molewaterplein 50, Rotterdam 3015 GE, The Netherlands; 2 Department of Population Health, London School of Hygiene and Tropical Medicine, Keppel Street, London WC1E 7HT, UK

**Keywords:** Clustered data, Concordance, Discrimination, Meta-analysis, Prediction, Risk model

## Abstract

**Background:**

The discriminative ability of a risk model is often measured by Harrell’s concordance-index (c-index). The c-index estimates for two randomly chosen subjects the probability that the model predicts a higher risk for the subject with poorer outcome (concordance probability). When data are clustered, as in multicenter data, two types of concordance are distinguished: concordance in subjects from the same cluster (within-cluster concordance probability) and concordance in subjects from different clusters (between-cluster concordance probability). We argue that the within-cluster concordance probability is most relevant when a risk model supports decisions within clusters (e.g. who should be treated in a particular center). We aimed to explore different approaches to estimate the within-cluster concordance probability in clustered data.

**Methods:**

We used data of the CRASH trial (2,081 patients clustered in 35 centers) to develop a risk model for mortality after traumatic brain injury. To assess the discriminative ability of the risk model within centers we first calculated cluster-specific c-indexes. We then pooled the cluster-specific c-indexes into a summary estimate with different meta-analytical techniques. We considered fixed effect meta-analysis with different weights (equal; inverse variance; number of subjects, events or pairs) and random effects meta-analysis. We reflected on pooling the estimates on the log-odds scale rather than the probability scale.

**Results:**

The cluster-specific c-index varied substantially across centers (*IQR* = 0.70-0.81; *I*^*2*^ = 0.76 with 95% confidence interval 0.66 to 0.82). Summary estimates resulting from fixed effect meta-analysis ranged from 0.75 (equal weights) to 0.84 (inverse variance weights). With random effects meta-analysis – accounting for the observed heterogeneity in c-indexes across clusters – we estimated a mean of 0.77, a between-cluster variance of 0.0072 and a 95% prediction interval of 0.60 to 0.95. The normality assumptions for derivation of a prediction interval were better met on the probability than on the log-odds scale.

**Conclusion:**

When assessing the discriminative ability of risk models used to support decisions at cluster level we recommend meta-analysis of cluster-specific c-indexes. Particularly, random effects meta-analysis should be considered.

## Background

Assessing the performance of a risk model is of great practical importance. An essential aspect of model performance is separating subjects with good outcome from subjects with poor outcome (discrimination) [1]. The concordance probability is a commonly used measure of discrimination reflecting the association between model predictions and true outcomes [2,3]. For binary outcome data it is the probability that a randomly chosen subject from the event group has a higher predicted probability of having an event than a randomly chosen subject from the non-event group. For time-to-event outcome data it is the probability that, for a randomly chosen pair of subjects, the subject who experiences the event of interest earlier in time has a lower predicted value of the time to the occurrence of the event. For both kinds of outcome data the concordance probability is often estimated with Harrell’s concordance (c)-index [2].

In risk modelling, clustered data are frequently used. A typical example is multicenter patient data, i.e. data of patients who are treated in different centers with similar inclusion criteria across the centers. Patients treated in the same center are nevertheless more alike than patients from different centers. A comparable type of clustering may occur in patients treated in different countries or in patients treated by different caregivers in the same center. Similarly, in public health research the study population is often clustered in geographical regions like countries, municipalities or neighbourhoods. It has been suggested that clustering should be taken into account in the development of risk models to obtain unbiased estimates of predictor effects [4]. This can be done by using a multilevel logistic regression model for binary outcomes or a frailty model for time-to-event outcomes [5,6].

It would be natural to take clustering also into account when measuring the performance of a risk model. For multilevel models, it has been proposed to consider the concordance probability of subjects within the same cluster (within-cluster concordance probability) separately from the concordance probability of subjects in different clusters (between-cluster concordance probability) [7,8]. We propose using the within-cluster concordance probability when risk models are used to support decisions within clusters, e.g. in clinical practice where decisions on interventions are commonly taken within centers. A valuable risk model should then be able to separate subjects within the same cluster into those with good outcome and poor outcome. We consider the within-cluster concordance probability more relevant in this context than the between-cluster or overall concordance probability.

Here, we aimed to estimate the within-cluster concordance probability from clustered data. We explored different meta-analytic methods for pooling cluster-specific concordance probability estimates with an illustration in predicting mortality among patients suffering from traumatic brain injury.

## Methods

### Mortality in traumatic brain injury patients

We present a case study of predicting mortality after Traumatic Brain Injury (TBI). Risk models using baseline characteristics provide adequate discrimination between patients with good and poor 6-month outcomes after TBI [9,10]. We used patients enrolled in the Medical Research Council Corticosteroid Randomisation after Significant Head Injury [11] trial (registration ISRCTN74459797, http://www.controlled-trials.com/), who were recruited between 1999 and 2004. This was a large international double-blind, randomized placebo-controlled trial of the effect of early administration of a 48-h infusion of methylprednisolone on outcome after head injury. The trial included 10,008 adults clustered in 239 centers with Glasgow Coma Scale (GCS) [12] Total Score ≤ 14, who were enrolled within 8 hours after injury. By design the patient inclusion criteria were equal in all 239 centers.

We considered patients with moderate or severe brain injury (GCS Total Score ≤ 12) and observed 6-month Glasgow Outcome Scale (GOS) [13]. Patients who were treated in one of 35 European centers with more than 5 patients experiencing the event (*n* = 2,081), were used to assess the discriminative ability of a prediction model developed with data from 35 centers. Patients who were treated in one of 21 Asian centers with more than 5 patients experiencing the event (*n* = 1,421) were used to assess the discriminative ability at external validation.

We used a Cox proportional hazards model with age, GCS Motor Score and pupil reactivity as covariates similar to previously developed risk models [9,10]. We modelled center with a Gamma frailty (random effect) to account for heterogeneity in mortality among centers. We estimated parameters on the European selection of patients with the R package survival [14,15]. As center effect estimates are unavailable when using a risk model in new centers, we calculated individual risk predictions applying the Gamma frailty mean of 1 for each patient.

### Cluster-specific concordance probabilities

We estimated the concordance probability within each cluster by Harrell’s c-index [2], i.e. the proportion of all usable pairs of subjects in which the predictions are concordant with the outcomes. A pair of subjects is usable if we can determine the ordering of their outcomes. For binary outcomes, pairs of subjects are usable if one of the subjects had an event and the other did not. For time-to-event outcomes, pairs of subjects are usable if their failure times are not equal and at least the smallest failure time is uncensored. For a usable subject pair the predictions are concordant with the outcomes if the ordering of the predictions is equal to the ordering of the outcomes. Values of the c-index close to 0.5 indicate that the model does not perform much better than a coin-flip in predicting which subject of a randomly chosen pair will have a better outcome. Values of the c-index near 1 indicate that the model is almost perfectly able to predict which subject of a randomly chosen pair will have a favourable outcome. We estimated the variances of the cluster-specific c-indexes with a method proposed by Quade [16]. Formulas are provided in Appendix 1.

### Pooling cluster-specific concordance probability estimates

The within-cluster concordance probability *C*_*w*_ can be estimated by pooling the cluster-specific concordance probability estimates into a weighted average. Previously, the cluster-specific concordance probability estimates were pooled with the number of usable subject pairs as weights [7,8]. Here, we define eight different ways for pooling of cluster-specific estimates – both on the probability scale and on the log-odds scale – based on fixed effect meta-analysis and random effects meta-analysis.

We consider a dataset with subjects in *K* clusters. Let *m*_*k*_ be the number of subjects and *e*_*k*_ be the number of events in cluster *k*. We denote the number of usable subject pairs – pairs of subjects for whom we can determine the ordering of their outcomes – in cluster *k* by *n*_*k*_. The cluster-specific concordance probability estimate for cluster *k* is denoted by ${\hat{C}}_{k}$ with sampling variance estimate ${\hat{\sigma }}_{k}^{2}$.

#### Fixed effect meta-analysis

Fixed effect meta-analysis assumes that one common within-cluster concordance probability *C*_*W*_ exists that applies to all clusters. The observed cluster-specific estimates vary only because of chance created from sampling subjects. Fixed effect meta-analysis with cluster weights *w*_*k*_ results in:

(1) $${\hat{C}}_{W}=\frac{\sum_{k}w_{k}{\hat{C}}_{k}}{\sum_{k}w_{k}}\;\mathrm{with}\;{\hat{\sigma }}_{{\hat{C}}_{W}}^{2}=\frac{\sum_{k}w_{k}^{2}{\hat{\sigma }}_{k}^{2}}{(\sum_{k}w_{k}){}^{2}}$$

The simplest approach would be to apply equal weights, *w*_*k*_ = 1/*K* for each cluster (method 1). This estimator is quite naive when the cluster size varies, because small clusters are given the same weight as large clusters and information about the precision of the cluster-specific estimates is ignored. Heuristic choices of weights taking the cluster size into account are the number of subjects, *w*_*k*_ = *m*_*k*_ (method 2), or the number of events, *w*_*k*_ = *e*_*k*_ (method 3). Analogous to the definition of the c-index a fourth option is the number of usable subject pairs as weights, *w*_*k*_ = *n*_*k*_ (method 4). The pooled estimate is then equal to the proportion of all usable within-cluster subject pairs in which the predictions and outcomes are concordant. Another choice of meta-analysis weights are the inverse variances, $w_{k}=1/{\hat{\sigma }}_{k}^{2}$ (method 5). These weights express the precision of the cluster-specific estimates and are commonly used in meta-analysis of study-specific treatment effects.

#### Random effects meta-analysis

In our context a random effects meta-analysis considers that the cluster-specific estimates vary not only because of sampling variability but also because of differences in true concordance probabilities. This is appropriate for high values of *I*^*2*^[17]. *I*^*2*^ measures the proportion of variability in cluster-specific estimates that is due to between-cluster heterogeneity rather than chance. Random effects meta-analysis assumes that cluster-specific concordance probabilities *C*_*k*_ are distributed about mean *μ* with between-cluster variance *τ*^2^, with the observed ${\hat{C}}_{k}$ normally distributed about *C*_*k*_ with sampling variance $\sigma_{k}^{2}$. The mean within-cluster concordance probability estimate $\hat{\mu }$ is the average of the cluster-specific estimates with the inverse variances as weights (method 6):

(2) $$\begin{array}{l} \hat{\mu }=\frac{\sum_{k}w_{k}{\hat{C}}_{k}}{\sum_{k}w_{k}},{\hat{\sigma }}_{\hat{\mu }}^{2}=\frac{\sum_{k}w_{k}^{2}\left({\hat{\sigma }}_{k}^{2}+{\hat{\tau }}^{2}\right)}{{\left(\sum_{k}w_{k}\right)}^{2}}\\ =\frac{1}{\sum_{k}w_{k}}\mathrm{with}\;w_{k}=1/\left({\hat{\sigma }}_{k}^{2}+{\hat{\tau }}^{2}\right) \end{array}$$

For estimation of the between-cluster variance *τ*^2^ we used the DerSimonian and Laird [18] method. Alternative estimators for *τ*^2^ can be found in DerSimonian and Kacker [19].

**Figure 1:**
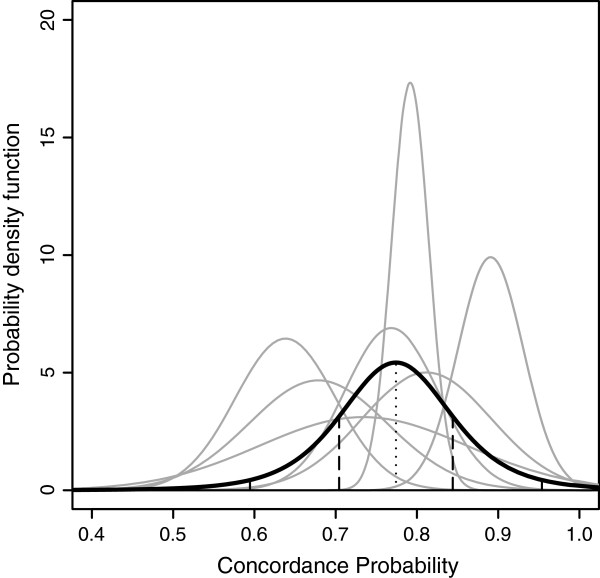
**Example of random effects meta-analysis of concordance probability estimates in 7 clusters.** Cluster-specific estimates are in grey. Under the assumption of normally distributed cluster-specific concordance probabilities, the predictive distribution resulting from a random effects meta-analysis is in black. The mean, the mean ± one standard deviation and the 2.5 and 97.5 percentiles of the predictive distribution are plotted with vertical lines.

With the additional assumption of normally distributed *C*_*k*_ we can derive a prediction interval for the within-cluster concordance probability *C*_*W*_ in a new or unspecified cluster [20]. If *τ*^2^ were known, then $\hat{\mu }\sim N\left(\mu ,{\hat{\sigma }}_{\hat{\mu }}^{2}\right)$ and *C*_*W*_ ~ *N*(*μ*, *τ*^2^) imply (assuming independence of *C*_*W*_ and $\hat{\mu }$ given *μ* ) that $C_{W}-\hat{\mu }\sim N\left(0,\tau^{2}+{\hat{\sigma }}_{\hat{\mu }}^{2}\right)$. Hence the within-cluster concordance probability *C*_*W*_ in a new cluster is normally distributed, with mean $\hat{\mu }$ and variance $\tau^{2}+{\hat{\sigma }}_{\hat{\mu }}^{2}$ (Figure 1). Since *τ*^2^ is estimated, we assume $\frac{C_{W}-\hat{\mu }}{\sqrt{{\hat{\tau }}^{2}+{\hat{\sigma }}_{\hat{\mu }}^{2}}}$ to take a more conservative t-distribution with *K* - 2 degrees of freedom instead of the standard normal distribution [20]. Thus, a 95% prediction interval of the within-cluster concordance probability *C*_*W*_ in an unspecified cluster can be approximated by: $\hat{\mu }\pm t_{K-2}^{0.975}\sqrt{{\hat{\tau }}^{2}+{\hat{\sigma }}_{\hat{\mu }}^{2}}$ with $t_{K-2}^{0.975}$ denoting the 97.5% percentile of the t-distribution with *K* - 2 degrees of freedom.

#### Meta-analysis scale

When calculating a prediction interval of the within-cluster concordance probability *C*_*W*_, Riley et al [21] advised to perform a random effects meta-analysis on a scale that helps meet the normality assumption for the random effects. When the normality assumption of the random effects model holds, the *C*_*k*_ are normally distributed with mean *μ* and variance $\tau^{2}+\sigma_{k}^{2}$. As a consequence, the standardized residuals *z*_*k*_ defined below should approximately have a standard normal distribution:

(3) $$z_{k}=\left({\hat{C}}_{k}-\hat{\mu }\right)/\sqrt{{\hat{\tau }}^{2}+{\hat{\sigma }}_{k}^{2}}$$

To consider if the normality assumption is valid we used a normal probability plot of *z*_*k*_ and applied the Shapiro-Wilk test to *z*_*k*_[22]. In a normal probability plot *z*_*k*_ is plotted against a theoretical normal distribution in such a way that the points should form an approximate straight line. Departures from this straight line indicate departures from normality. The Shapiro-Wilk test returns the probability of obtaining the test-statistic as least as extreme as the observed one, under the null-hypothesis that *z*_*k*_ are normally distributed (p-value). When the p-value is above significance level α, say 5%, the null hypothesis that *z*_*k*_ is normally distributed is not rejected.

Since the concordance probability is restricted to [0, 1] the normality assumption of random effects meta-analysis may be violated. We considered inverse variance weighted meta-analysis on the log-odds scale as an alternative approach (methods 7 and 8 for fixed effect and random effects meta-analysis respectively). The resulting estimators for the within-cluster concordance probability are defined in Appendix 2. The normality assumption on log-odds scale was again assessed by the normal probability plot and the Shapiro-Wilk test.

Table 1 contains a summary of the eight pooling methodologies described above. For all the meta-analyses we used the R package rmeta [14,23].

**Table 1:** Overview of the 8 methods for pooling of cluster-specific concordance probability estimates

	**Fixed effect meta-analysis**	**Random effects meta-analysis**
	**Assuming the same true (logit) concordance probability within each cluster**	**Assuming variation in true (logit) concordance probabilities across clusters**
**Probability scale**		
Meta-analysis of cluster-specific estimates of the concordance probability	1. Equal weight for each cluster	6. Inverse of the sum of the cluster-specific sampling variance estimate and the between-cluster variance estimate
	2. Number of subjects in the cluster	
	3. Number of subjects in the cluster with an event	
	4. Number of usable subject pairs within the cluster	
	5. Inverse of the cluster-specific sampling variance estimate	
**Log-odds scale**		
Meta-analysis of cluster-specific estimates of the logit concordance probability	7. Inverse of the cluster-specific sampling variance estimate on log-odds scale	8. Inverse of the sum of the cluster-specific sampling variance estimate on log-odds scale and the between-cluster variance estimate on log-odds scale

## Results

The European patients were slightly older in comparison with the Asian patients (median age 36 vs. 31 years) and were more likely to have the worst GCS Motor Score of 1, i.e. no motor response (21% versus 4%) compared to the Asian patients (Table 2). However, 6 month mortality was lower in the European patients (27%) than in the Asian patients (35%).

**Table 2:** Patient characteristics in selected European and Asian centers

**Characteristic**	**Measure or Category**	**Europe**		**Asia**	
**Age (years)**	Median (25–75 percentile)	36	(24–53)	31	(22–43)
**GCS Motor score**	No response (1)	445	(21%)	55	(4%)
	Extension (2)	134	(6%)	96	(7%)
	Abnormal flexion (3)	176	(8%)	124	(9%)
	Normal flexion (4)	321	(15%)	261	(18%)
	Localizes/obeys (5/6)	1,005	(48%)	885	(62%)
**Pupil reactivity**	No pupil reacted	291	(14%)	129	(9%)
	One pupil reacted	123	(6%)	117	(8%)
	Both pupils reacted	1,667	(80%)	1,175	(83%)
**Six-month mortality**	Dead	553	(27%)	495	(35%)
**Patients**	Total	2,081		1,421	
**Centers**	Total	35		21	
**Patients per center**	Median (25–75 percentile)	33	(21–64)	34	(20–66)

We found that 6-month mortality was clearly associated with higher age, worse GCS Motor Score and less pupil reactivity (Table 3). Heterogeneity in mortality among European centers was substantial as indicated by the hazard ratio of 1.7 for the 75 percentile versus the 25 percentile of the random center effect, based on the quartiles of the Gamma frailty distribution with mean 1 and variance estimate 0.146.

**Table 3:** Associations between predictors and 6-month mortality in European centers

**Characteristic**	**Level**	**HR (95 % CI)**	
**Age (years)**	47 versus 23*	2.1	(1.9-2.4)
**GCS Motor score**	No response (1)	3.1	(2.4-4.0)
	Extension (2)	2.8	(2.0-3.8)
	Abnormal flexion (3)	2.4	(1.7-3.2)
	Normal flexion (4)	1.5	(1.1-2.0)
	Localizes/obeys (5/6)	1.0	(ref)
**Pupil reactivity**	No pupil reacted	2.8	(2.3-3.5)
	One pupil reacted	1.7	(1.2-2.3)
	Both pupils reacted	1.0	(ref)
**Center random effect**	75 versus 25 percentile	1.7	

*Interquartile range.

**Figure 2:**
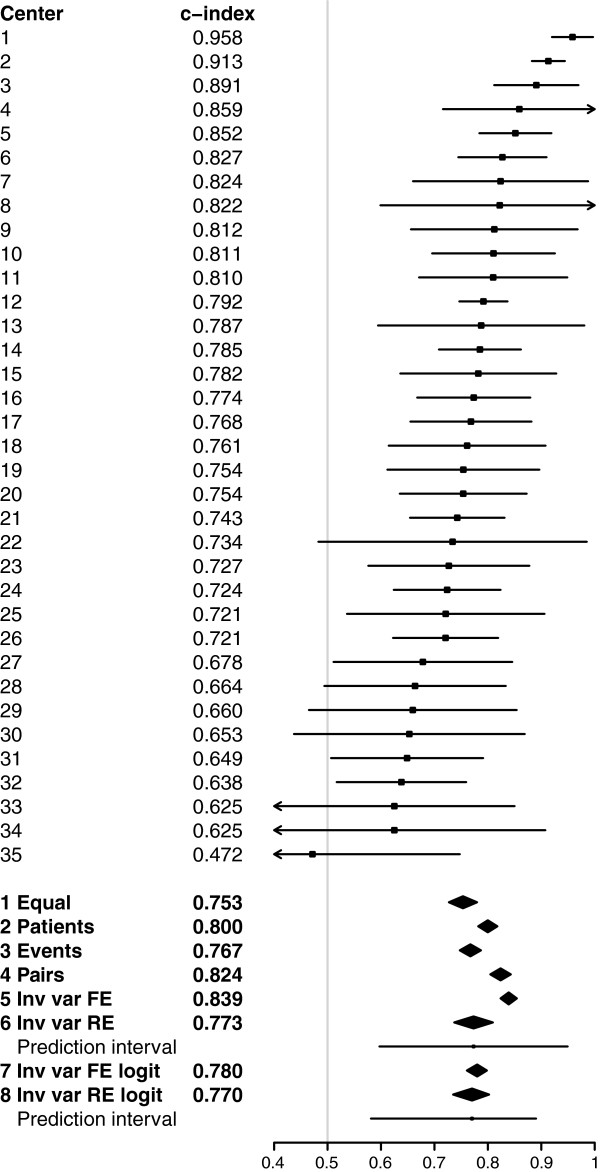
**Center-specific and pooled concordance probability estimates with 95% confidence intervals for European centers.** For pooled estimates based on random effects meta-analysis a 95% prediction interval for the concordance probability is presented by a horizontal line. 1 Equal = Fixed effect meta-analysis with equal weights; 2 Patients = Fixed effect meta-analysis with number of patients as weights; 3 Events = Fixed effect meta-analysis with number of events as weights; 4 Pairs = Fixed effect meta-analysis with number of usable patient pairs as weights; 5 Inv var FE = Fixed effect meta-analysis with inverse variance weights; 6 Inv var RE = Random effects meta-analysis with inverse variance weights; 7 Inv var FE logit = Fixed effect meta-analysis with inverse variance weights on log-odds scale; 8 Inv var RE logit = Random effects meta-analysis with inverse variance weights on log-odds scale.

Among European centers (overall c-index 0.80) the c-indexes varied substantially with an interquartile range of 0.70 to 0.81 (Figure 2). Pooled concordance probability estimates resulting from fixed effect meta-analysis ranged from 0.75 (equal weights) to 0.84 (inverse variance weights). Random effects meta-analysis (method 6) led to a mean concordance probability estimate $\hat{\mu }=0.77$, a between-cluster variance estimate ${\hat{\tau }}^{2}=0.0072$ and a wide 95% prediction interval (0.60 to 0.95) reflecting the strong heterogeneity in the cluster-specific concordance probabilities (*I*^*2*^ = 0.76 with 95% confidence interval 0.66 to 0.82). Random effects meta-analysis on log-odds scale (method 8) led to similar results, but with a somewhat smaller asymmetric prediction interval (0.58 to 0.89).

**Figure 3:**
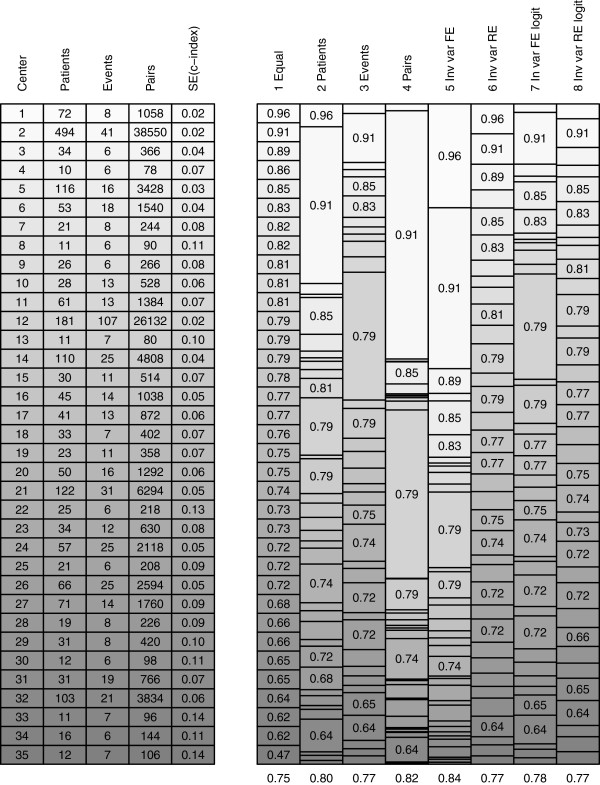
**Meta-analysis pooling weights for European centers.** For methods 1 to 8 the weights are represented by the height of the bars on the right hand side of the Figure. C-indexes are printed in the bars if the cluster weight was at least equal to the average weight. 1 Equal = Fixed effect meta-analysis with equal weights; 2 Patients = Fixed effect meta-analysis with number of patients as weights; 3 Events = Fixed effect meta-analysis with number of events as weights; 4 Pairs = Fixed effect meta-analysis with number of usable patient pairs as weights; 5 Inv var FE = Fixed effect meta-analysis with inverse variance weights; 6 Inv var RE = Random effects meta-analysis with inverse variance weights; 7 Inv var FE logit = Fixed effect meta-analysis with inverse variance weights on log-odds scale; 8 Inv var RE logit = Random effects meta-analysis with inverse variance weights on log-odds scale.

Large differences in pooling weights, together with heterogeneity in the cluster-specific concordance probabilities, led to very different pooled estimates. We analysed the pooling weights to explain the differences in pooled estimates (Figure 3). The patient-weighted estimate was dominated by center 2 with 494 of the 2,081 patients. The event-weighted estimate was dominated by center 12 with 107 out of 553 events. The patient-pair-weighted estimate was heavily determined by both center 2 and center 12 as the number of usable patient pairs is related to the number of patients times the number of events. The fixed effect inverse-variance weighted estimate was also strongly influenced by centers with high number of patients or events, because the standard errors of the cluster-specific estimates depend heavily on the number of patients and events. Furthermore, the fixed effect inverse-variance weighted estimate was upwardly influenced by center 1 as a result of the small standard error relative to the small number of patients and events. The random effects inverse-variance weighted estimate was much less dominated by particular centers and close to the equally weighted estimate because of the large amount of heterogeneity. The standard error on the log-odds scale increased with increasing c-index according to Equation 10 in Appendix 2 and therefore put less weight on the centers with a high concordance probability estimate resulting in lower pooled estimates. The large standard errors for centers with high c-index also decreased the heterogeneity (*I*^*2*^ = 0.61 with 95% confidence interval 0.44 to 0.73) on the log-odds scale resulting in more similar weights for fixed effect and random effects meta-analysis.

**Figure 4:**
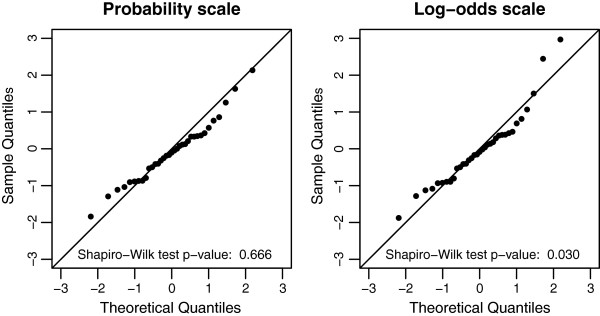
**Normal probability plot of standardized residuals on probability scale and log-odds scale (European centers).** Shapiro-Wilk test results are printed at the bottom of each plot.

To check the validity of the normality assumption in the random effects meta-analyses, we calculated standardized residuals (Equation 3), both on the probability and the log-odds scale. The standardized residuals better fitted to the standard normal distribution on the probability scale than on the log-odds scale (Figure 4, p-values for rejection of the normality null hypothesis of 0.666 on probability scale and of 0.030 on log-odds scale).

**Figure 5:**
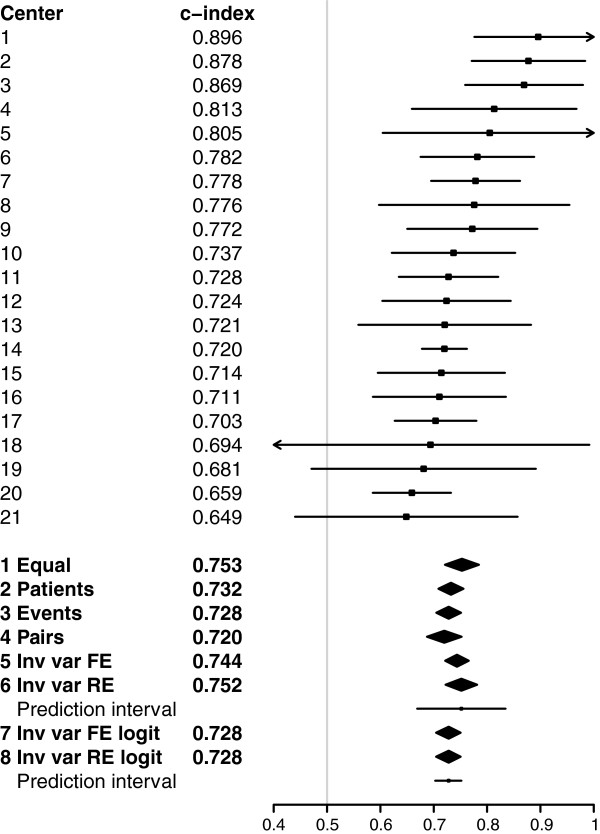
**Center-specific and pooled concordance probability estimates with 95% confidence intervals for Asian centers (external validation).** For pooled estimates based on random effects meta-analysis (methods 6 and 8) a 95% prediction interval for the concordance probability is presented by a horizontal line. 1 Equal = Fixed effect meta-analysis with equal weights; 2 Patients = Fixed effect meta-analysis with number of patients as weights; 3 Events = Fixed effect meta-analysis with number of events as weights; 4 Pairs = Fixed effect meta-analysis with number of usable patient pairs as weights; 5 Inv var FE = Fixed effect meta-analysis with inverse variance weights; 6 Inv var RE = Random effects meta-analysis with inverse variance weights; 7 Inv var FE logit = Fixed effect meta-analysis with inverse variance weights on log-odds scale; 8 Inv var RE logit = Random effects meta-analysis with inverse variance weights on log-odds scale.

To illustrate the comparison in an external validation setting, we repeated the analysis of the within-cluster concordance probability in Asian centers with the same risk model (Figure 5). Among Asian clusters (overall c-index 0.74) the c-indexes varied less (*IQR* 0.71-0.78), which was reflected in a lower proportion of variation among clusters that is due to heterogeneity rather than chance (*I*^*2*^ = 0.32 with 95% confidence interval 0 to 0.60). As a result, different pooling methodologies led to more similar pooled estimates, because differences in cluster weights have less impact when cluster-specific estimates are more alike. Based on random effects meta-analysis, estimates of the mean within-cluster concordance probability and the between-cluster variance were $\hat{\mu }=0.75$ and ${\hat{\tau }}^{2}=0.0013$ respectively. The resulting prediction interval (0.67 to 0.83) was much smaller than for the European clusters. The heterogeneity disappeared on the log-odds scale (*I*^*2*^ = 0) leading to equal estimates by fixed effect and random effects meta-analysis.

## Discussion

We studied how to assess the discriminative ability of risk models in clustered data. The within-cluster concordance probability is an important measure for risk models when these models are used to support decisions on interventions within the clusters. The within-cluster concordance probability can be estimated by pooling cluster-specific concordance probability estimates (e.g. c-indexes) with a meta-analysis, similar to pooling of study-specific treatment effect estimates. We considered different pooling strategies (Table 1) and recommend random effects meta-analysis in case of substantial variability – beyond chance – of the concordance probability across clusters [20,21]. To decide if the meta-analysis should be undertaken on the probability scale or the log-odds scale we suggest considering the normality assumptions on both scales by normal probability plots and Shapiro-Wilk tests of the standardized residuals.

The illustration of predicting 6-month mortality after TBI prompted the use of random effects meta-analysis because of the strong difference – beyond chance – in concordance probability among centers. This was clearly visualized by the forest plot in Figure 2. Random effects meta-analysis results can be summarized by the mean concordance probability and a 95% prediction interval for possible values of the concordance probability. By definition, these results give insight into the variation of the discriminative ability among centers as opposed to fixed effect meta-analysis results [20,21]. By comparing normal probability plots and Shapiro-Wilk test results based on the standardized residuals we concluded the random effects meta-analysis results on probability scale to be the most appropriate (Figure 4). Although the methodology is illustrated with time-to-event outcomes of traumatic brain injury patients, it is also applicable to binary outcomes.

Even if a risk model contains regression coefficients that are optimal for the data in each cluster, differences in case mix may lead to different concordance probabilities across clusters [24]. Furthermore, predictor effects may vary because of cluster-specific circumstances, also leading to different cluster-specific concordance probabilities. Given the variability beyond chance in our case study, we consider a random effects meta-analysis of the cluster-specific c-indexes as most appropriate.

The assumption of random effects meta-analysis is that underlying concordance probabilities among clusters are exchangeable, i.e. cluster-specific concordance probabilities are expected to be non-identical, yet identically distributed [20]. If part of the variation can be explained by cluster characteristics, a meta-regression – assuming partial exchangeability – of the concordance probability estimates with cluster characteristics as covariates is preferable.

We chose to analyse the concordance probability as it is the most commonly used measure of discriminative ability of a risk model. However, the same logic of pooling cluster-specific performance measure estimates can be applied to any other performance measure, like the discrimination slope, the explained variation (*R*^*2*^) or the Brier score [25].

We used Harrell’s c-index to estimate cluster-specific concordance probabilities together with Quade’s formula for the cluster-specific variances of the c-index [2,16]. The same methodology of pooling cluster-specific performance measure estimates can be applied to other concordance probability estimators and its variances. Other estimators for the concordance probability in time-to-event data can be found in Gönen and Heller [26] and Uno et al [27]. These estimators are especially favourable when censoring varies by cluster as they are shown to be less sensitive to censoring distributions. Other variance estimators are described by Hanley and McNeil [28], and DeLong et al [29] for binary outcome data and by Nam and D'Agostino [30] and Pencina and D'Agostino [3] for time-to-event outcome data. The variance of the concordance probability estimate can also be estimated with a bootstrap procedure [31].

## Conclusion

We recommend meta-analysis of cluster-specific c-indexes when assessing discriminative ability of risk models used to support decisions at cluster level. Particularly, random effects meta-analysis should be considered as it allows for and provides insight into the variability of the concordance probability among clusters.

## Appendix 1

The concordance probability is defined as the probability that a randomly chosen subject pair with different outcomes is concordant. For a randomly chosen subject pair (*i*, *j*) with outcomes *Y*_*i*_ and *Y*_*j*_ and model predictions ${\hat{Y}}_{i}$ and ${\hat{Y}}_{j}$ the concordance probability *C* is:

(4) $$C=\mathrm{Pr}\left(\left({\hat{Y}}_{i}<{\hat{Y}}_{j}\right|Y_{i}<Y_{j}\right)$$

Harrell’s c-index [2] estimates the concordance probability by the proportion of all usable pairs of subjects (*n*_*u*_) in which the predictions and outcomes are concordant (*n*_*c*_), with tied predictions (*n*_*t*_) counted as 1/2:

(5) $$\hat{C}=\frac{n_{c}+n_{t}/2}{n_{u}}$$

For binary outcomes *y*, pairs of subjects are usable if one of the subjects had an event and the other did not. The number of usable subject pairs *n*_*u*_, the number of concordant subject pairs *n*_*c*_ and the number of tied subject pairs *n*_*t*_ are:

(6) $$\begin{array}{l} n_{u}=\sum_{i}\sum_{j}I\left(y_{i}<y_{j}\right)\\ n_{c}=\sum_{i}\sum_{j}I\left(y_{i}<y_{j}\;\mathrm{and}\;{\hat{y}}_{i}<{\hat{y}}_{j}\right)\\ n_{t}=\sum_{i}\sum_{j}I\left(y_{i}<y_{j}\;\mathrm{and}\;{\hat{y}}_{i}={\hat{y}}_{j}\right) \end{array}$$

For time-to-event outcomes *y*, pairs of subjects are usable if their survival times are not equal and at least the smallest survival time is uncensored. We have to add the restriction that the smallest observation *y*_*i*_ of each subject pair is uncensored, denoted by *δ*_*i*_ = 1:

(7) $$\begin{array}{l} n_{u}=\sum_{i}\sum_{j}I\left(y_{i}<y_{j}\;\mathrm{and}\;\delta_{i}=1\right)\\ n_{c}=\sum_{i}\sum_{j}I\left(y_{i}<y_{j}\;\mathrm{and}\;\delta_{i}=1\;\mathrm{and}\;{\hat{y}}_{i}<{\hat{y}}_{j}\right)\\ n_{t}=\sum_{i}\sum_{j}I\left(y_{i}<y_{j}\;\mathrm{and}\;\delta_{i}=1\;\mathrm{and}\;{\hat{y}}_{i}={\hat{y}}_{j}\right) \end{array}$$

The variance of the c-index can be estimated according to Quade [16]:

(8) $${\hat{\sigma }}_{\hat{C}}^{2}=\frac{\sum n_{u,i}^{2}{\left(\sum n_{c-d,i}\right)}^{2}-2\sum n_{u,i}\sum n_{c-d,i}\sum n_{u,i}n_{c-d,i}+{\left(\sum n_{u,i}\right)}^{2}\sum n_{c-d,i}^{2}}{{\left(\sum n_{u,i}\right)}^{4}}$$

All summations over *i* with *n*_*u,i*_ and *n*_*c-d,i*_ the number of usable and the number of concordant minus discordant subject pairs of which subject *i* is one:

(9) $$\begin{array}{l} \begin{array}{l} n_{u,i}=\sum_{j}I\left(y_{i}<y_{j}\;\mathrm{and}\;\delta_{i}=1\right)\\ n_{c,i}=\sum_{j}I\left(y_{i}<y_{j}\;\mathrm{and}\;\delta_{i}=1\;\mathrm{and}\;{\hat{y}}_{i}<{\hat{y}}_{j}\right) \end{array}\\ n_{d,i}=\sum_{j}I\left(y_{i}<y_{j}\;\mathrm{and}\;\delta_{i}=1\;\mathrm{and}\;{\hat{y}}_{i}>{\hat{y}}_{j}\right)\\ n_{c-d,i}=n_{c,i}-n_{d,i} \end{array}$$

## Appendix 2

Based on the delta method, a variance estimator for the logit of the c-index is:

(10) $$\mathrm{var}\left(\log\mathrm{it}\left(\hat{C}\right)\right)=\mathrm{var}\left(\log\left(\frac{\hat{C}}{1-\hat{C}}\right)\right)=\frac{\mathrm{var}\left(\hat{C}\right)}{{\left(\hat{C}\left(1-\hat{C}\right)\right)}^{2}}$$

We used this variance estimator to perform a meta-analysis on log-odds scale. The pooling weights (method 7) for a fixed effect inverse variance meta-analysis on log-odds scale are:

(11) $$w_{k}={\left[\frac{{\hat{\sigma }}_{k}^{2}}{{\left({\hat{C}}_{k}\left(1-{\hat{C}}_{k}\right)\right)}^{2}}\right]}^{-1}$$

The pooling weights (method 8) for a random effects inverse variance meta-analysis on log-odds scale are:

(12) $$w_{k}={\left[\frac{{\hat{\sigma }}_{k}^{2}}{{\left({\hat{C}}_{k}\left(1-{\hat{C}}_{k}\right)\right)}^{2}}+{\hat{\tau }}^{2}\right]}^{-1}$$

The resulting pooled estimates together with confidence and prediction intervals are transformed back to probability scale.

## Abbreviations

c-index: Concordance-index; CRASH: Corticosteroid randomisation after significant head injury; GCS: Glasgow coma scale; GOS: Glasgow outcome scale; IQR: Interquartile range.

## Competing interests

The authors declare that they have no competing interests.

## Authors’ contributions

DK, ES and YV designed the study. PP participated in the collection of data and organisation of the databases from which this manuscript was developed. DK and YV analysed the data and wrote the first draft of the manuscript. All authors contributed to writing the manuscript and read and approved the final manuscript.

## Pre-publication history

The pre-publication history for this paper can be accessed here:

http://www.biomedcentral.com/1471-2288/14/5/prepub
